# Open Multiligament Reconstruction for Concomitant Patellofemoral and Type IV Tibiofemoral Dislocation and Bucket‐Handle Medial Meniscus Injury

**DOI:** 10.1002/atn2.70192

**Published:** 2026-07-19

**Authors:** Sina Javidmehr, Armin Akbarzadeh, Robert F. LaPrade

**Affiliations:** ^1^ Department of Orthopaedic Surgery Sina Hospital Tehran University of Medical Sciences Tehran Iran; ^2^ Orthopedic & Rehabilitation Research Center, Shiraz University of Medical Sciences Shiraz Iran; ^3^ Department of Orthopedic Surgery School of Medicine Shiraz University of Medical Sciences Shiraz Iran; ^4^ Twin Cities Orthopedics Edina Minnesota U.S.A.

## Abstract

A knee dislocation combined with a patellofemoral dislocation is an extremely rare and complex injury that reflects severe multidirectional instability and extensive soft‐tissue disruption. We show an open single‐stage anatomic reconstruction method for a patient with type IV tibiofemoral dislocation, patellofemoral dislocation, and bucket‐handle medial meniscus tear. A midline parapatellar approach provided direct access to both the medial and lateral compartments, allowing for the identification of all ligament attachments and the repair of the meniscal tear. Cruciate, collateral, posterolateral corner, and medial patellofemoral ligament tears were reconstructed using tibialis posterior allografts. To avoid tunnel convergence, common femoral tunnels were drilled for the posterior cruciate ligament‐medial collateral ligament and anterior cruciate ligament‐fibular collateral ligament complexes with an additional femoral tunnel for the popliteofibular ligament. The open approach allowed for an anatomical restoration of all major stabilizers, while minimizing multiple skin incisions and avoiding the complications related to arthroscopic fluid extravasation. Early reconstruction was indicated due to gross instability of both tibiofemoral and patellofemoral joints and the associated meniscal tear. This technique provides a practical and safe alternative for comprehensive anatomic reconstruction in selected cases of complex knee dislocation with severe capsular disruption.

## Graphical abstract

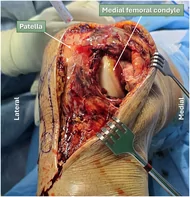

**VIDEO 1:** The physical examination and our open multiligament reconstruction technique is illustrated (Right side). (ACL, anterior cruciate ligament; FCL, fibular collateral ligament; MPFL, medial patellofemoral ligament; PCL, posterior cruciate ligament; PFL, popliteofibular ligament; POL, posterior oblique ligament; sMCL, superficial medial collateral ligament.) Video content can be viewed at https://doi.org/10.1002/atn2.70192.

Knee dislocation is a rare injury but is associated with disruption of multiple ligamentous structures and a high risk of neurovascular injury. Schenck type IV injuries, which involve both cruciate and collateral ligaments, is the most unstable pattern and frequently require complex reconstruction to restore tibiofemoral stability.^1^ Concomitant patellofemoral dislocation and extensive capsuloligamentous disruption further increase the complexity of management.^2^

Most multiligament knee injuries are currently treated using arthroscopic‐assisted or hybrid reconstruction techniques; however, in the setting of severe soft‐tissue disruption and capsular rupture, open reconstruction may offer advantages, including direct visualization and anatomic reconstruction of the structures. Also, it may avoid risk of compartment syndrome by the reduction of operation time and avoidance of fluid extravasation. Published reports have shown that single‐stage anatomic reconstruction can restore stability and function in selected multiligament injury patterns, although the optimal surgical strategy remains debated.^1^

In this study, we describe a complex case of Schenck's type IV tibiofemoral and patellofemoral dislocation, and bucket‐handle meniscus injury that was referred from another center after 2 weeks. The multiligament injuries were reconstructed in a single stage using an open approach.

## SURGICAL TECHNIQUE

Because of the potential for significant vascular injury in this injury pattern, vascular status was evaluated by checking distal pulse and computed tomographical angiography, both of which confirmed normal vascular flow.

**FIGURE 1:**
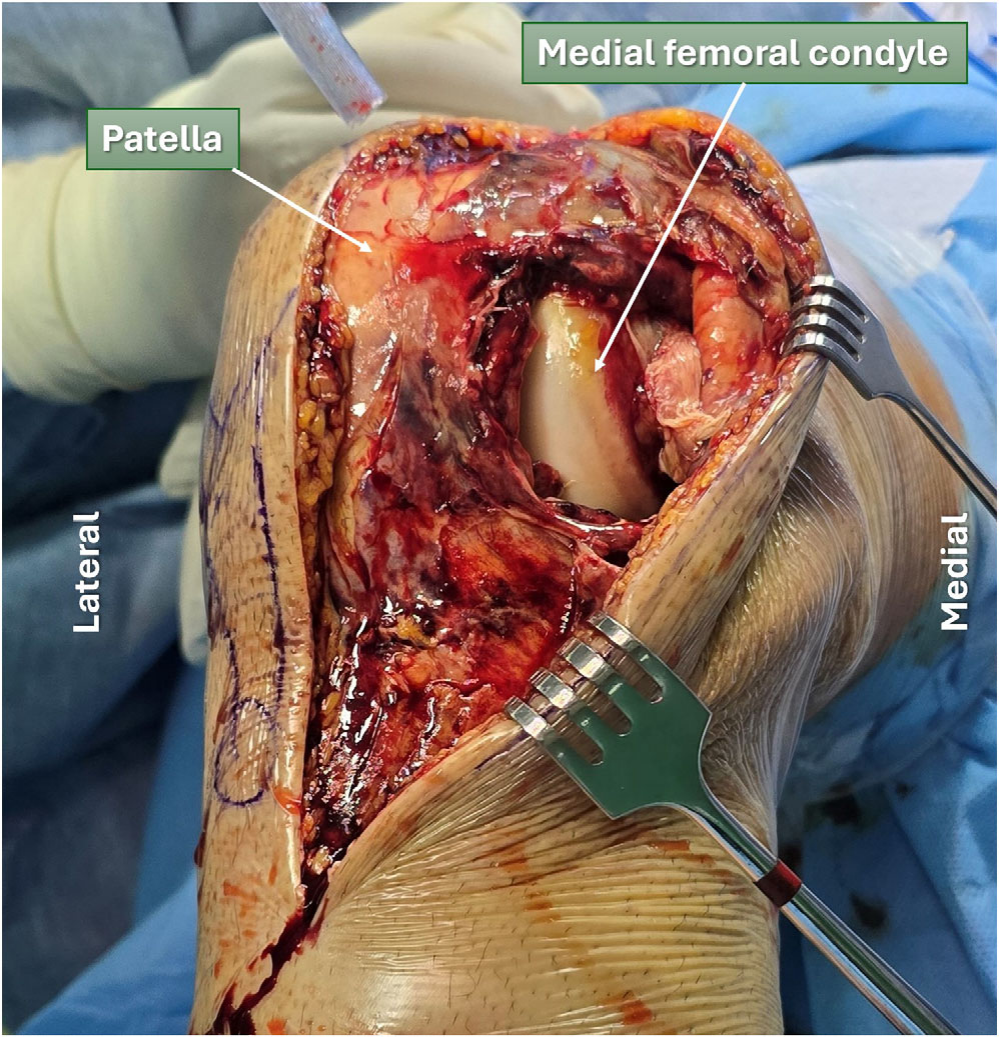
The medial retinaculum rupture was seen after a midline incision on the right knee in flexed position.

Through a midline incision, the skin and the subcutaneous tissues were opened, exposing the medial retinaculum, which showed extensive injury (Figure 1).

**FIGURE 2:**
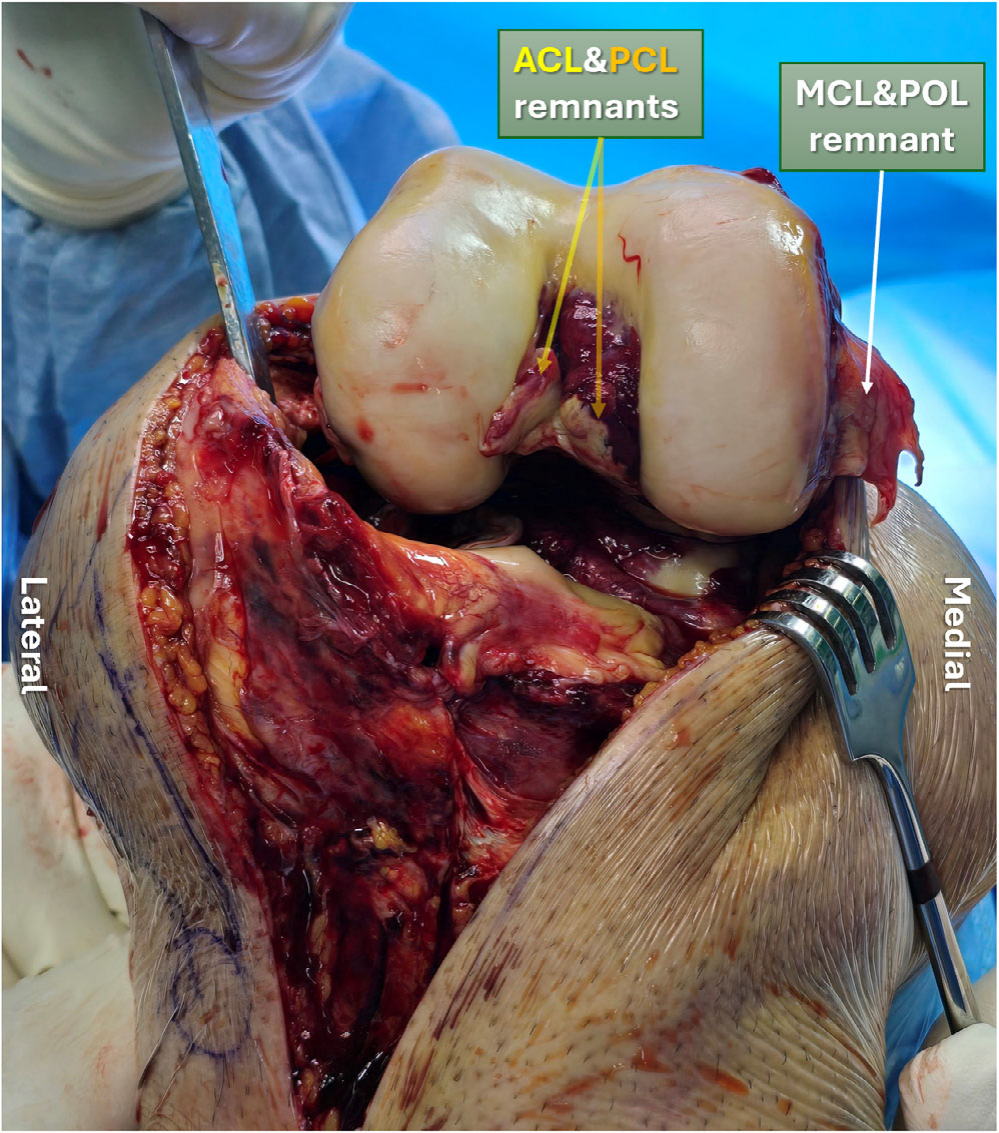
The proximal tibia was dislocated posteriorly after extending the medial retinaculum rupture and converting it to a medial parapatellar approach. The stumps of ruptured ACL, PCL, and medial stabilizers (including sMCL and POL) are seen at the right femur. (ACL, anterior cruciate ligament; MCL, medial collateral ligament; PCL, posterior cruciate ligament; POL, posterior oblique ligament; sMCL, superficial medial collateral ligament.)

The ruptured medial retinaculum was extended superiorly and inferiorly to create a medial parapatellar approach. After retracting the patella with the patellar and quadriceps tendons, the proximal tibia was identified subluxated posteriorly, and the distal femur was visualized. As shown in Figure 2, both the anterior cruciate ligament (ACL) and the posterior cruciate ligament (PCL) were avulsed from their tibial origins.

**FIGURE 3:**
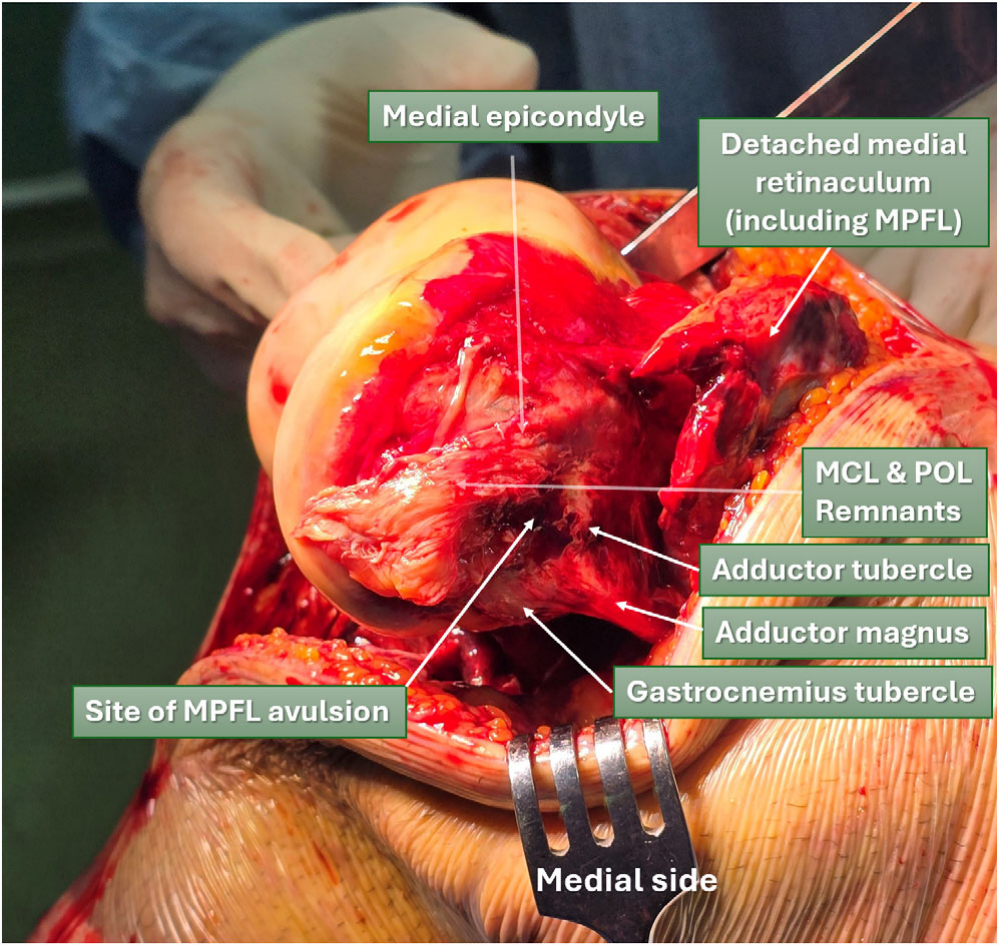
Intrasubstance rupture of all ligamentous attachments at the distal medial aspect of the right femur is seen. (MCL, medial collateral ligament; MPFL, medial patellofemoral ligament; POL, posterior oblique ligament.)

**FIGURE 4:**
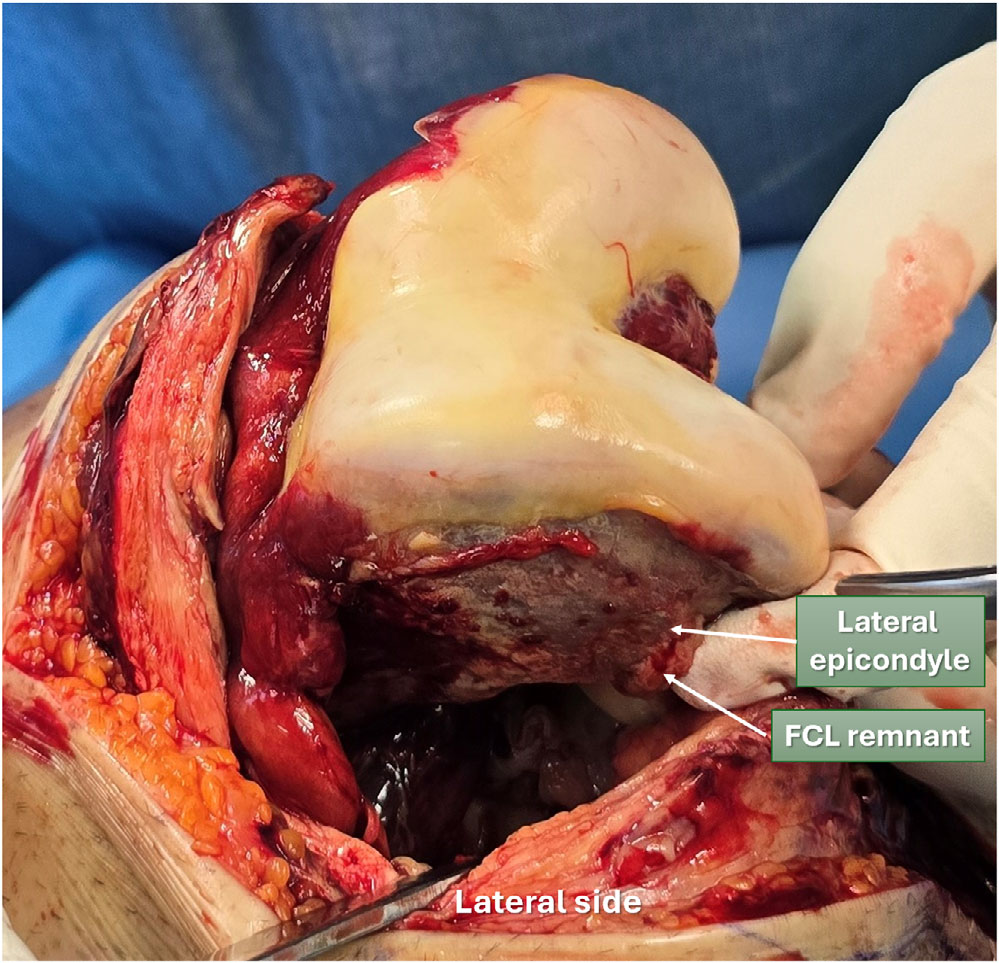
All ligamentous structures were avulsed from the lateral aspect of the right femur. (FCL, fibular collateral ligament.)

The femoral origins of the medial structures, including the superficial medial collateral ligament (sMCL), posterior oblique ligament (POL), medial patellofemoral ligament (MPFL), and the medial head of the gastrocnemius were ruptured (Figure 3). Similarly, the femoral origins of the lateral structures, including the fibular collateral ligament (FCL), popliteus tendon, anterolateral ligament, and the lateral head of the gastrocnemius were completely detached from bone (Figure 4). The medial meniscofemoral and meniscotibial ligaments were also torn, with the medial meniscus displaced into the intercondylar notch, although its roots remained intact.

### Femoral Reconstruction Tunnel Preparation

**FIGURE 5:**
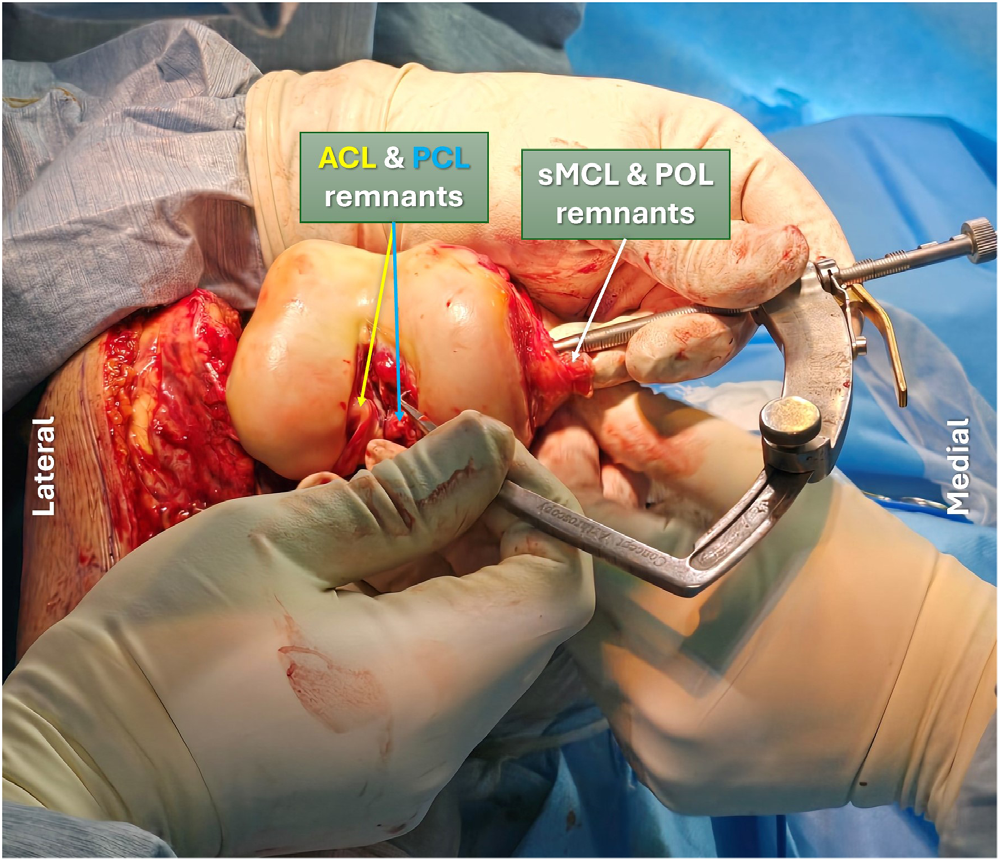
The direction of the common PCL‐MCL tunnel was identified by insertion of a guide pin from the femoral origin of sMCL to the stump of PCL femoral attachment, using the ACL jig (ConMed, Utica, NY) (Right side). (ACL, anterior cruciate ligament; PCL‐MCL, posterior cruciate ligament‐medial collateral ligament; POL, posterior oblique ligament; sMCL, superficial medial collateral ligament.)

**FIGURE 6:**
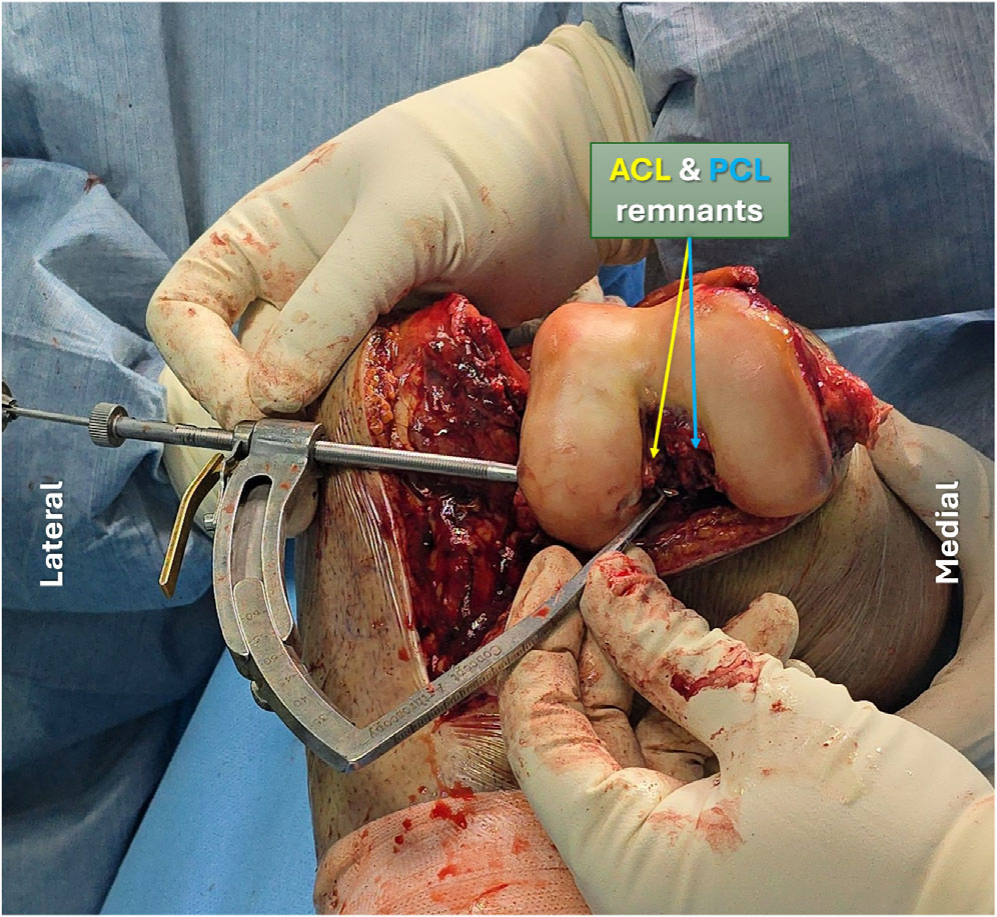
The direction of the common ACL‐FCL tunnel was identified by insertion of a guide pin from the femoral origin of FCL to the stump of ACL femoral attachment, using the ACL jig (ConMed, Utica, NY) (Right side). (ACL‐FCL, anterior cruciate ligament‐fibular collateral ligament; PCL, posterior cruciate ligament.)

To prevent tunnel convergence, a common femoral tunnel for the PCL and sMCL was prepared in the medial condyle, and another common femoral tunnel for the ACL and FCL in the lateral femoral condyle (Table 1). The anatomic origin of the sMCL (4.8 mm posterior and 3.2 mm proximal to the medial epicondyle)^3^ and the anterolateral bundle of PCL (7.4 mm from trochlear point and 11 mm from the medial arch point)^4^ were identified. Similarly, the origin of the FCL (3.1 mm posterior and 1.4 mm proximal to the lateral epicondyle)^5^ and the ACL stump (6.1 mm posterior to the lateral intercondylar ridge and 1.7 mm proximal to the bifurcate ridge) were identified at the lateral femoral condyle.^6^ Guide‐wires were inserted through mentioned landmarks using an ACL jig (ConMed, Utica, NY), and the common PCL‐MCL and ACL‐FCL tunnels were created (Figures 5 and 6).

**TABLE 1:** Pearls and Pitfalls

Pearls	Pitfalls
Identify anatomic footprints of all ligaments (ACL, PCL, MCL, FCL, POL, MPFL, PFL) before drilling to prevent tunnel malposition	Failure to confirm anatomical landmarks may result in nonanatomic graft positioning and postoperative instability or stiffness
Create common femoral tunnels (PCL‐MCL and ACL‐FCL) to reduce the number of drill holes and avoid tunnel convergence	Creating multiple tunnels instead of applying common tunnels and anchor sutures when feasible may lead to tunnel convergence and weakening of the distal femoral bone
Sequentially tension and fix grafts at appropriate flexion angles: PCL‐MCL at 90°, ACL‐PLC in full extension, MPFL at 30°	Inappropriate graft tensioning in an improper knee position can result in overconstraint or residual laxity of the knee
Protect the common peroneal nerve during fibular head exposure and tunnel preparation	Inadequate identification of the peroneal nerve may cause iatrogenic nerve injury
Repair and reduce the meniscus before ligament reconstruction to restore joint congruency and avoid later graft impingement	Neglecting the bucket‐handle meniscus tear before graft passage can compromise reduction and make further meniscus repair difficult due to limited access
Direct the PFL tunnel anterosuperiorly to prevent convergence with ACL‐FCL tunnel	A misaligned PFL tunnel can cause convergence with other tunnels or penetration to the femoral notch
Maintain soft‐tissue coverage over the reconstructed MPFL to prevent impingement on the femoral condyle	An uncovered graft may rub against the femoral condyle, limit motion, and cause failure

ACL, anterior cruciate ligament; ACL‐FCL, anterior cruciate ligament‐fibular collateral ligament; ACL‐PLC, anterior cruciate ligament‐posterolateral corner; FCL, fibular collateral ligament; MCL, medial collateral ligament; MPFL, medial patellofemoral ligament; PCL, posterior cruciate ligament; PCL‐MCL, posterior cruciate ligament‐medial collateral ligament; PFL, popliteofibular ligament; POL, posterior oblique ligament; sMCL, superficial medial collateral ligament.

**FIGURE 7:**
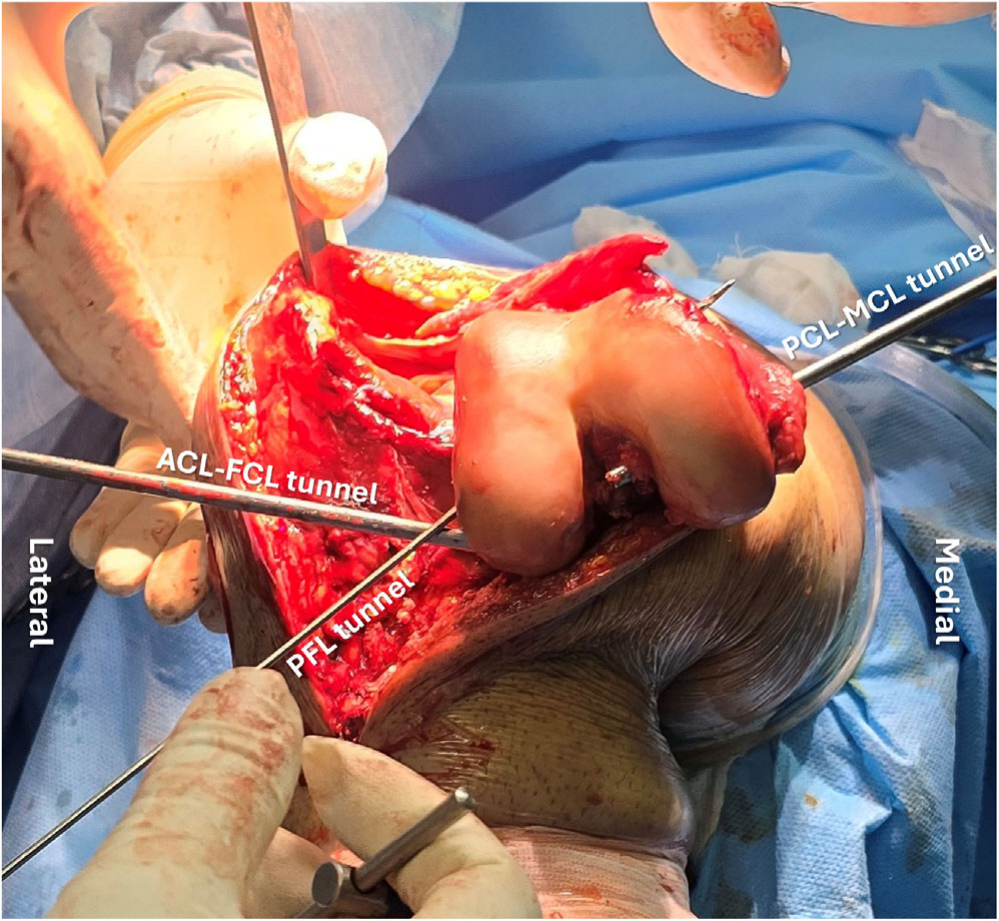
The PFL tunnel was prepared at its anatomical place, anterior and distal to the ACL‐PLC tunnel, using the ACL jig (ConMed, Utica, NY). The direction of the 3 femoral tunnels is shown (Right side). (ACL‐PLC, anterior cruciate ligament‐posterolateral corner; FCL, fibular collateral ligament; PCL, posterior cruciate ligament; PFL, popliteofibular ligament; MCL, medial collateral ligament.)

Both tunnels were reamed to a diameter of 8 mm to accommodate double‐layered tibialis posterior allografts. A third femoral tunnel was then created 18.5 mm anterior and distal to the FCL tunnel^4^ with a 7 mm reamer and 2 cm depth for the popliteofibular ligament tunnel as originally described by Arciero. The trajectory of this tunnel was directed anterosuperiorly to avoid convergence with other tunnels (Figure 7). Looped nylon sutures were passed through all tunnels for subsequent graft passage.

### Tibial Tunnel Preparation

**FIGURE 8:**
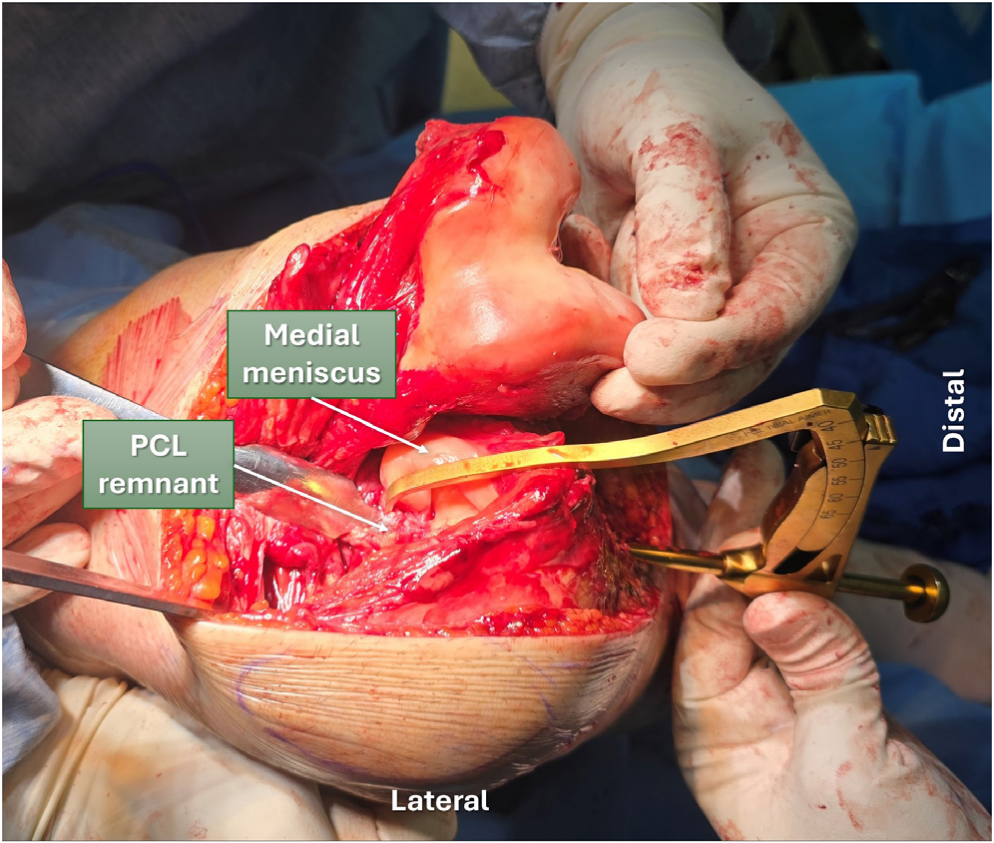
The tibial PCL tunnel is prepared at its anatomical footprint using the PCL jig (ConMed, Utica, NY) (Right side). (PCL, posterior cruciate ligament.)

**FIGURE 9:**
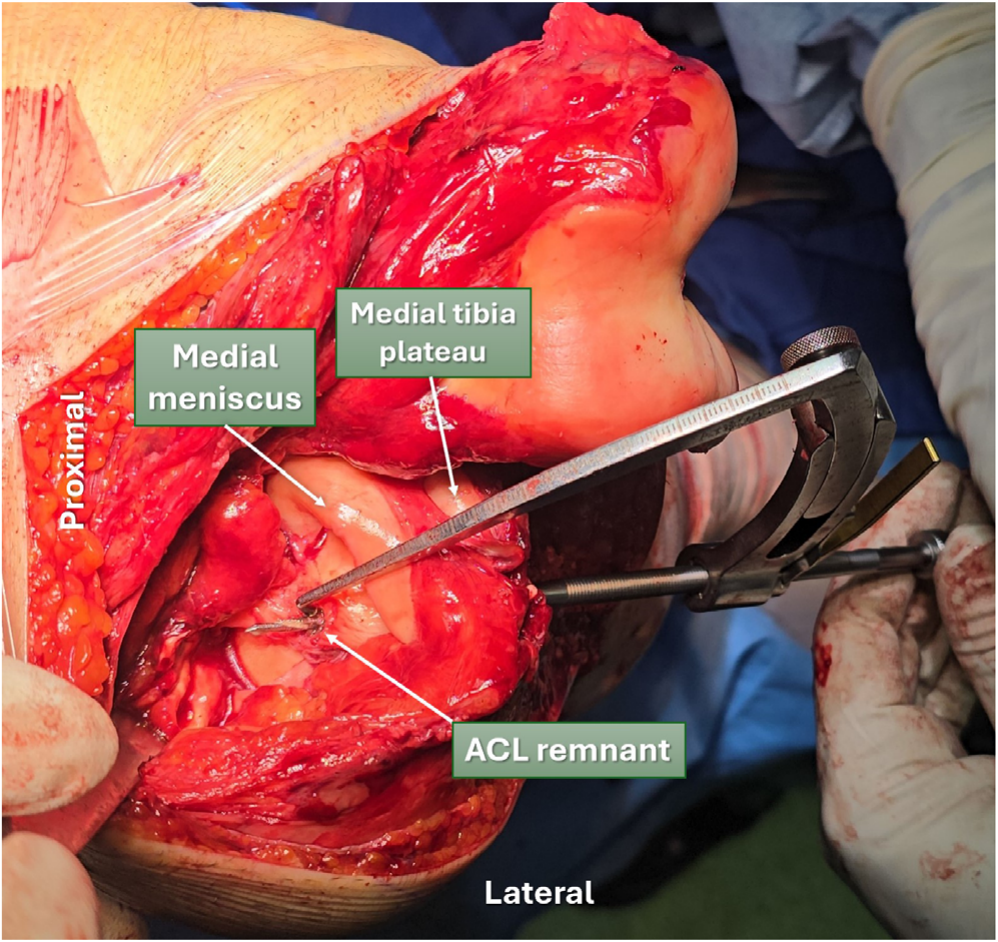
The tibial ACL tunnel is prepared at its anatomical footprint using the ACL jig (ConMed, Utica, NY) (Right side). (ACL, anterior cruciate ligament.)

**FIGURE 10:**
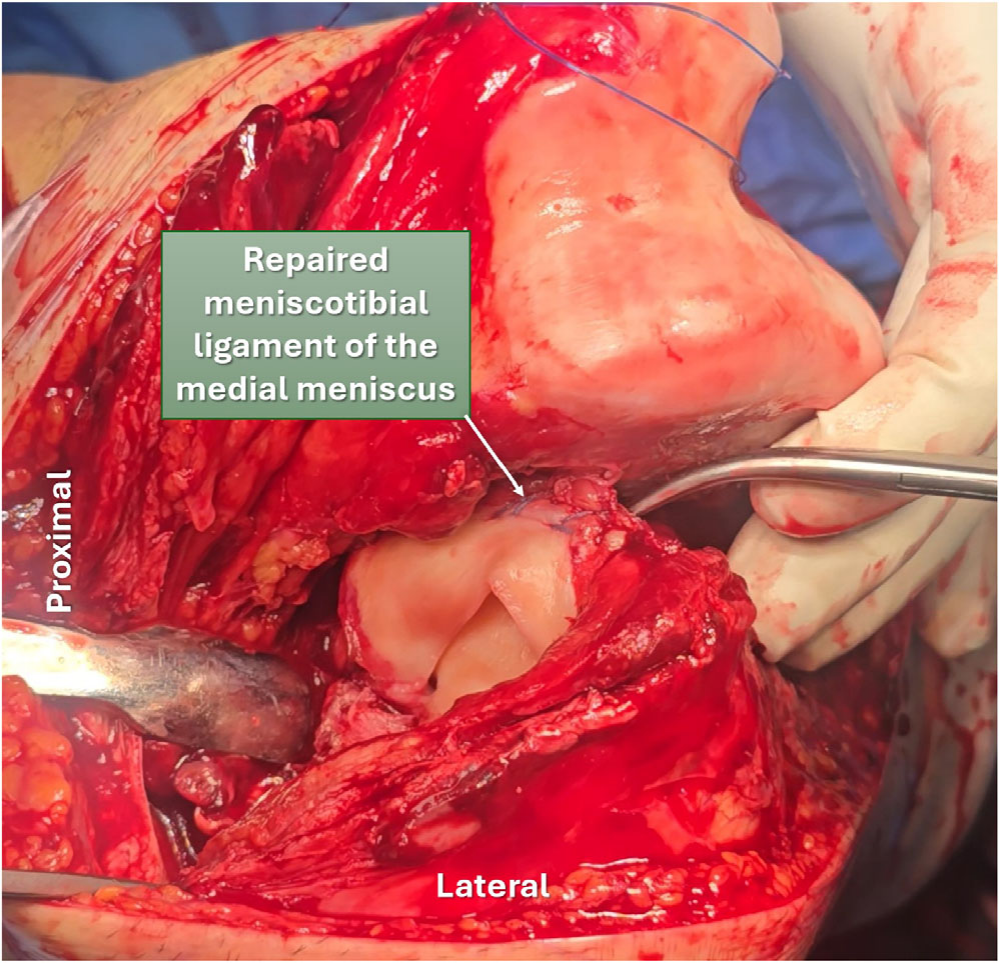
The medial meniscus was reduced in its anatomical site, and its meniscotibial ligament was repaired (Right side).

The tibial reconstruction tunnels were prepared using the anatomic tibial footprints of the ACL (7.5 mm medial to the anterior horn of the lateral meniscus)^5^ and PCL (at the medial side of the PCL facet, just anterosuperior to the bundle ridge)^3^ were identified, guide pins were inserted using their respective jigs (ConMed, Utica, NY), and the tunnels were reamed to 8 mm diameters (Figures 8 and 9). The sMCL tibial insertion was identified 6 cm distal to the joint line, and an 8 mm closed socket tunnel was prepared. Looped nylon sutures were passed through all tunnels for graft passage. Then, the medial meniscus was reduced and its meniscotibial ligaments were repaired (Figure 10).

### Fibular Tunnel Preparation

A separate 3 cm incision was made over the fibular head. The stability of the proximal tibiofibular joint was assessed and found to be stable; therefore, the posterolateral corner (PLC) was reconstructed using the Arciero technique. After exploration and protection of the common peroneal nerve, the insertion point of the FCL was identified 8 mm posterior to the anterior fibular border and 23 mm distal to the tip of the styloid. A guide wire was passed from this point toward the posterosuperior direction of the fibular head and reamed to 7 mm. A nylon suture was passed through the prepared tunnel for later graft passage.

### Knee Reduction and Cruciate and Collateral Ligament Fixation

**FIGURE 11:**
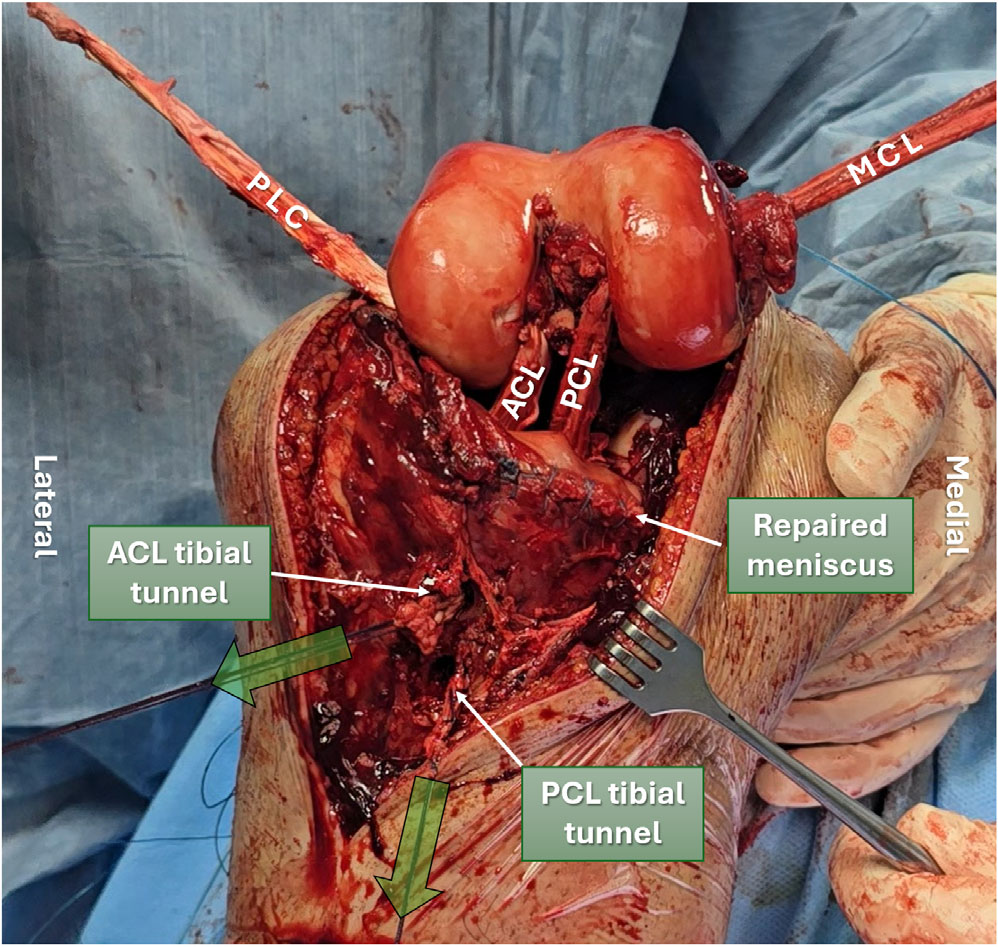
The tibiofemoral joint was reduced while applying traction on the free ends of the grafts (Right side). (ACL, anterior cruciate ligament; PCL, posterior cruciate ligament; PLC, posterolateral corner; MCL, medial collateral ligament.)

The PCL‐MCL graft was passed through the PCL tibial tunnel and then the medial femoral tunnel. The ACL‐PLC graft was passed through the ACL tibial tunnel and then the common ACL‐PLC femoral tunnel at the lateral femoral condyle. The femoral fixations of the grafts were carried out using interference screws (ConMed Linvatec, Largo, FL). The tibiofemoral joint was then reduced while traction was applied to the tibial graft ends (Figure 11). The tibial fixation of the PCL‐MCL graft was performed at 90° of flexion with a posterior‐to‐anterior force to correct posterior sagging. The tibial fixation of the ACL‐PLC graft was performed in full extension.

### MCL and POL Reconstruction

**FIGURE 12:**
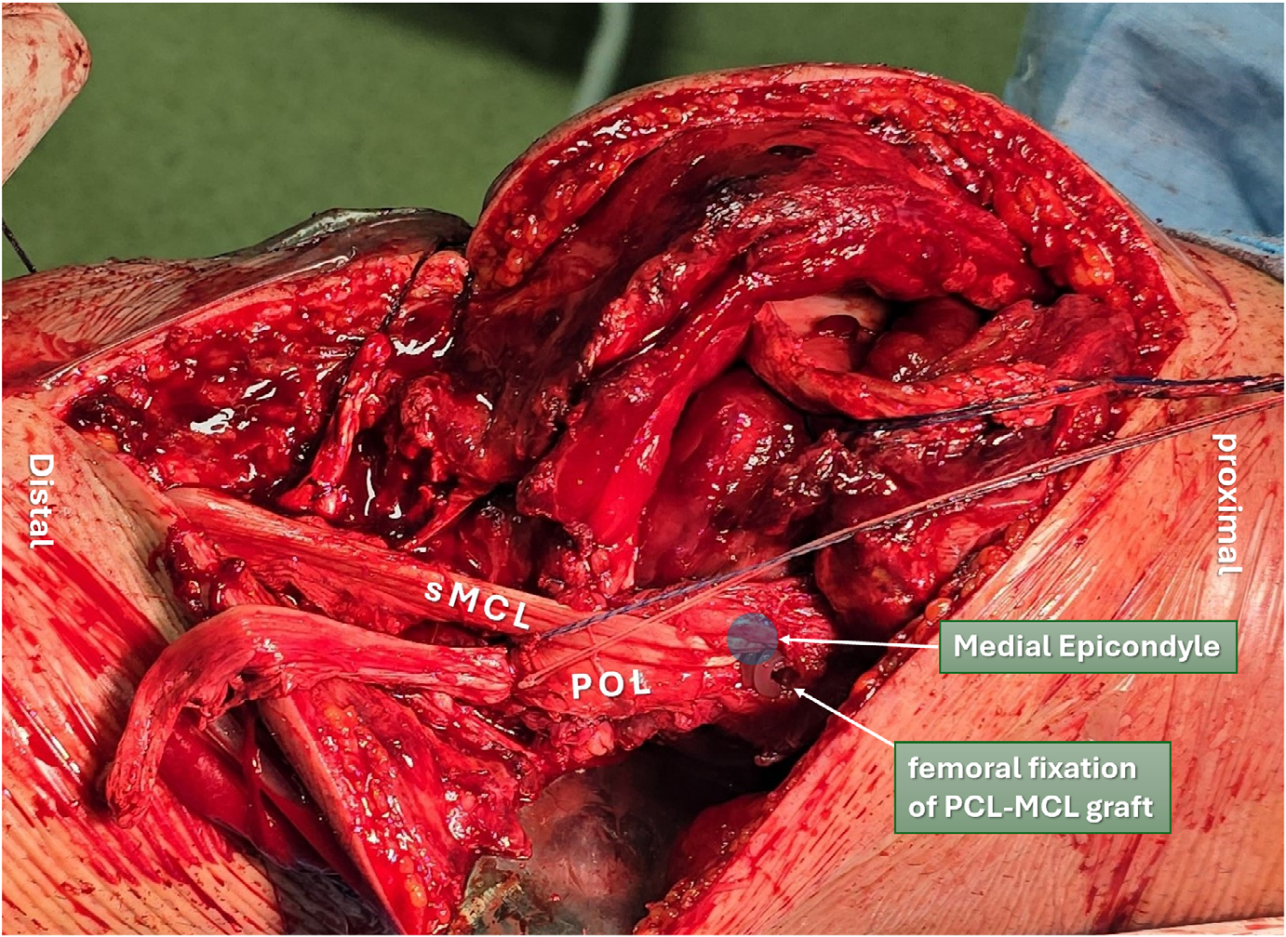
The reconstructed sMCL and POL by 2 strands of allograft, which originated from the PCL‐MCL tunnel 1 and 6 cm distal to the medial joint line and posteromedial tibial corner, respectively (Right side). (PCL‐MCL, posterior cruciate ligament‐medial collateral ligament; POL, posterior oblique ligament; sMCL, superficial medial collateral ligament.)

The free ends of the double‐layered PCL‐MCL graft were separately used to reconstruct the sMCL and POL. The anterior limb was fixed in the previously prepared sMCL tibial tunnel using an interference screw at 20° of flexion, neutral rotation, and mild varus reduction force. The reconstructed sMCL was also fixed 1 cm distal to the medial tibial plateau using an anchor suture to reproduce the proximal tibial attachment of the sMCL. The posterior limb was fixed to the posteromedial corner of the tibia to reconstruct the POL in a similar knee position, using a suture anchor (Pejvak Teb Pooya, Tehran, Iran) (Figure 12).

### PLC Reconstruction

**FIGURE 13:**
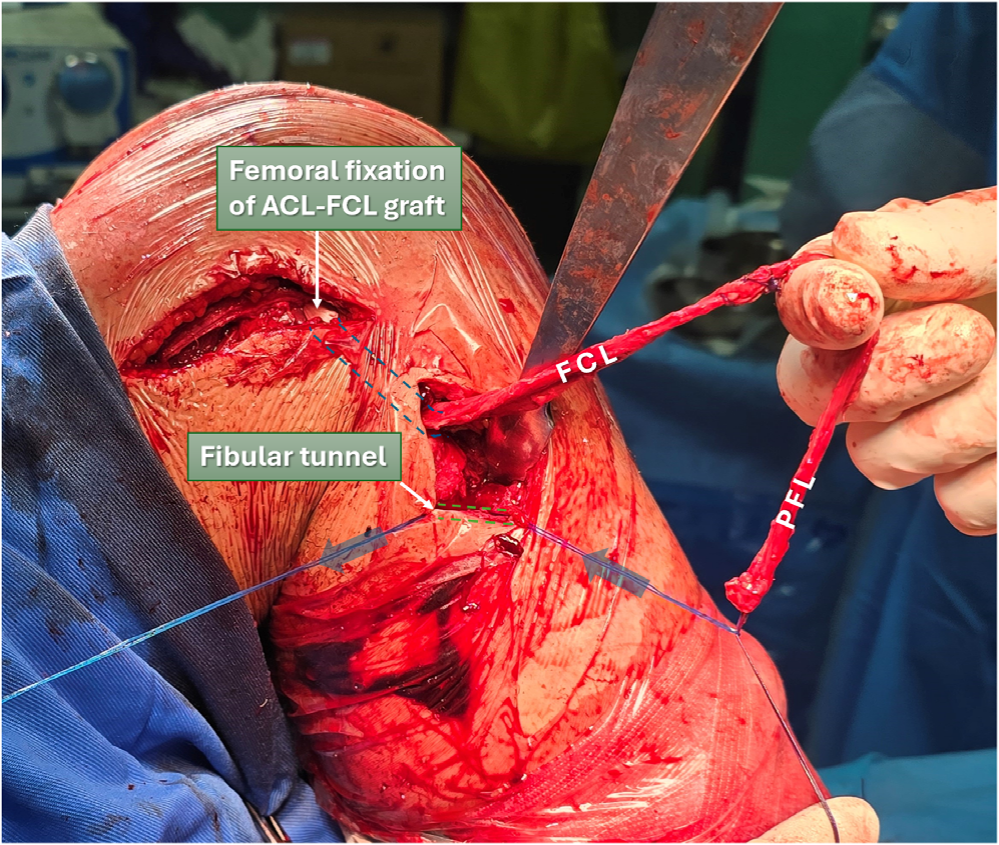
The PLC reconstruction. After femoral fixation of the ACL‐PLC graft, its free end was passed beneath the iliotibial band and through the prepared fibular tunnel from anterior to posterior (Right side). (ACL‐PLC, anterior cruciate ligament‐posterolateral corner; FCL, fibular collateral ligament; PFL, popliteofibular ligament; PLC, posterolateral corner.)

**FIGURE 14:**
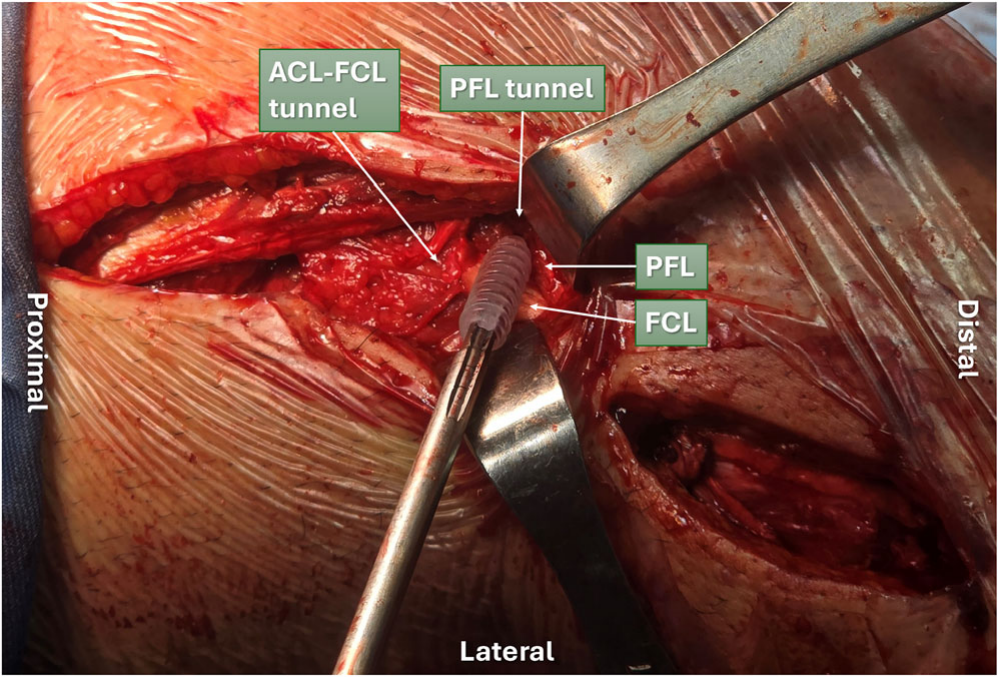
Fixation of ACL‐PLC graft to prepared PFL tunnel after passing it through the fibular tunnel and retrieving it anteriorly beneath the FCL using interference screw (ConMed Linvatec, Largo, FL) (Right side). (ACL‐PLC, anterior cruciate ligament‐posterolateral corner; FCL, fibular collateral ligament; PFL, popliteofibular ligament.)

One of the free ends of the double‐layered ACL‐PLC graft was cut and sutured to the other, creating a longer single‐strand graft for PLC reconstruction. Before suturing, we determined the appropriate graft length and ensured proper positioning of the sutured segment within the fibular tunnel. The graft was passed beneath the iliotibial band, retrieved through the lateral incision, and passed through the fibular tunnel from anterior to posterior (Figure 13). Both graft segments were then secured with an interference screw (ConMed Linvatec, Largo, FL). The free end of the graft was then returned to the site of femoral popliteus insertion, and passed beneath the reconstructed FCL. The graft was fixed in the popliteofibular ligament tunnel while the knee was in 30° of flexion, internal rotation, and slight valgus using an interference screw (ConMed Linvatec, Largo, FL) (Figure 14).

### MPFL Reconstruction

**FIGURE 15:**
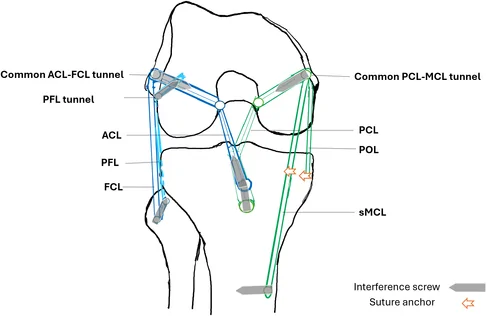
The anteroposterior schematic view of tibiofemoral joint ligamentous reconstruction (in 90° knee flexion) (Right side). (ACL, anterior cruciate ligament; FCL, fibular collateral ligament; PCL, posterior cruciate ligament; PFL, popliteofibular ligament; POL, posterior oblique ligament; sMCL, superficial medial collateral ligament.)

**FIGURE 16:**
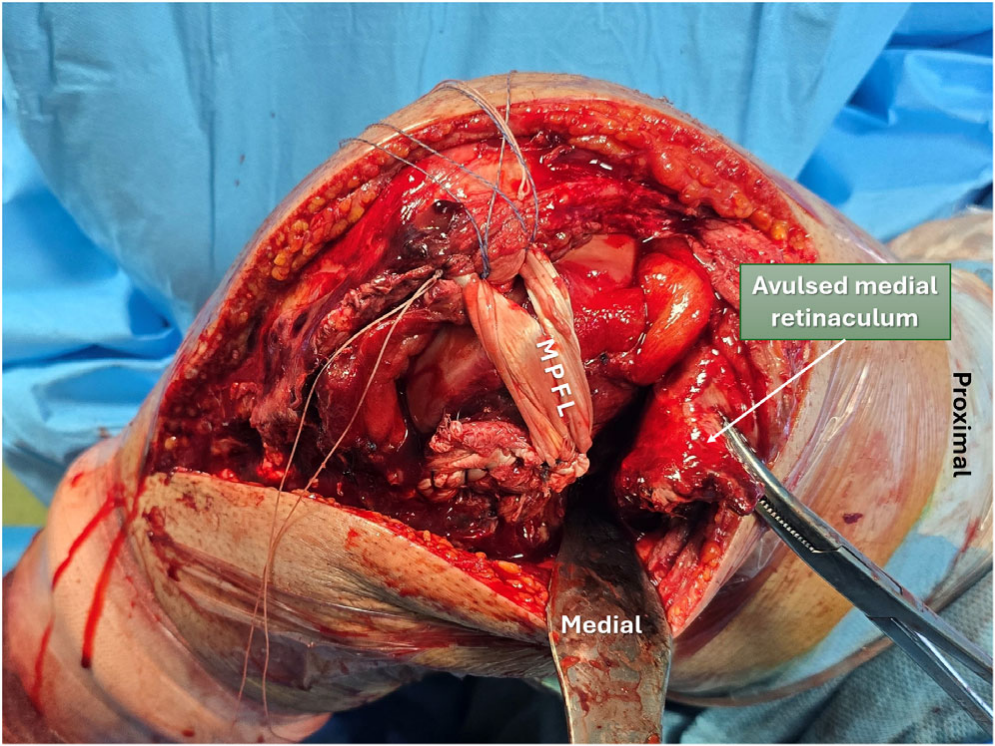
The MPFL reconstruction was done using 3 suture anchors (Pejvak Teb Pooya, Tehran, Iran) and a bent allograft, after medial patellar surface preparation (Right side). (MPFL, medial patellofemoral ligament.)

**FIGURE 17:**
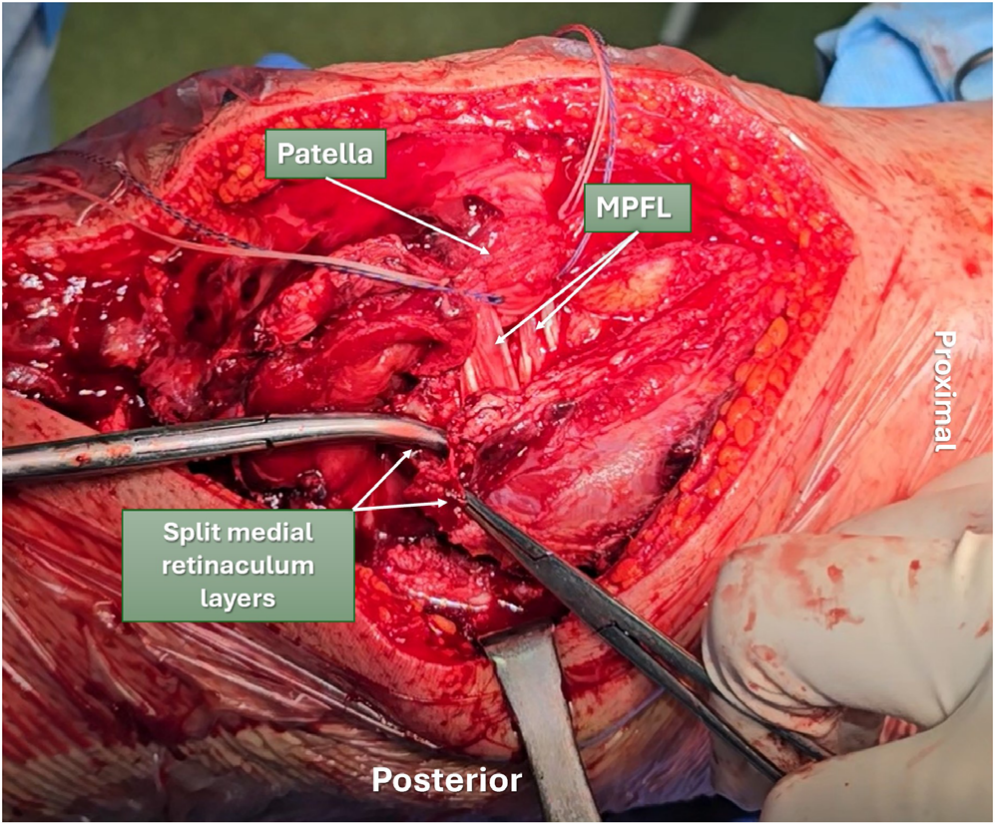
The medial retinaculum was split between the 2nd and 3rd layers, and the reconstructed MPFL was covered to avoid impingement with the femoral condyle during motion (Right side). (MPFL, medial patellofemoral ligament.)

**FIGURE 18:**
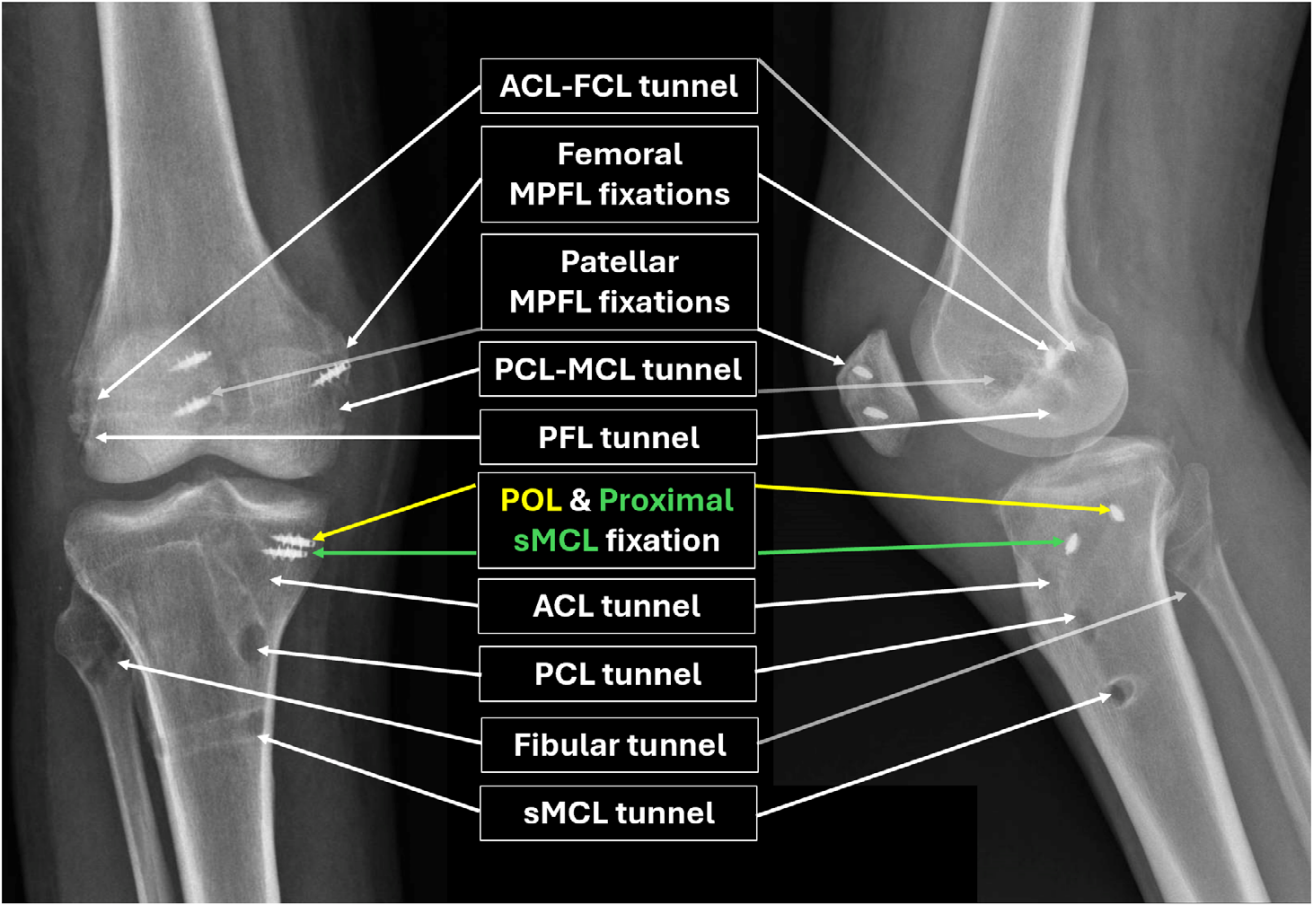
The postoperative X‐ray of the reconstructed knee (Right side). (ACL, anterior cruciate ligament; FCL, fibular collateral ligament; MPFL, medial patellofemoral ligament; PFL, popliteofibular ligament; PCL, posterior cruciate ligament; POL, posterior oblique ligament; sMCL, superficial medial collateral ligament.)

After reconstruction of the cruciate and collateral ligaments and stabilization of the tibiofemoral joint (Figure 15), the MPFL was reconstructed using another tibialis posterior allograft. The soft tissues were removed from the proximal medial surface of the patella using a curette. The midpoint of the doubled over allograft was fixed to the prepared medial patellar edge using 2 suture anchors (Pejvak Teb Pooya, Tehran, Iran). The medial retinaculum was opened between the 2nd and the 3rd layers. The free ends were passed through these layers and fixed to the anatomic footprint of the MPFL at the femur (8.9 mm proximal and 4.6 mm posterior to the medial epicondyle) using an anchor suture while the knee was flexed in 30° flexion and the patella was located at the center of the trochlear groove. (Figures 16 and 17). Postoperative X‐ray confirmed proper reduction of both the tibiofemoral and patellofemoral joints, with anatomic placement of tunnels and suture anchors (Pejvak Teb Pooya, Tehran, Iran) (Figure 18). The surgical technique is also illustrated in Video 1.

## DISCUSSION

Although knee dislocation type IV itself is not a prevalent injury, the simultaneous occurrence of patellofemoral dislocation has only been described in isolated case reports and small case series. This combination represents a high‐energy mechanism resulting in multidirectional instability and extensive soft‐tissue disruption involving both tibiofemoral and patellofemoral stabilizers.^2^ In our case, the coexistence of these injuries created gross instability of both the tibiofemoral and the patellofemoral joints. Also, the presence of gross tibiofemoral and patellofemoral instability, as well as a bucket‐handle medial meniscus tear, supported the decision for early anatomic reconstruction of all major stabilizers to restore stability, meniscal congruency, and patellar tracking. Single‐stage anatomic reconstruction is a promising and effective treatment for certain cases of multiligament knee injuries, including severe knee dislocations. When the soft‐tissue conditions are favorable, this approach can yield outcomes similar to those of staged procedures, without a significant increase in the complication rate, like stiffness or graft failure.^7^ Because of the extreme rarity of this injury pattern, there is no established treatment algorithm; however, immediate recognition, careful assessment of soft‐tissue and vascular status, and an anatomic reconstruction when feasible may offer the best chance for restoring joint function and preventing long‐term instability.^8^, ^9^, ^10^, ^11^

Although arthroscopic techniques are the choice for multiligament reconstruction, their application in complex injuries with capsular rupture and extensive soft‐tissue damage may be associated with prolonged operative time and fluid extravasation. In this case, complete disruption of the medial retinaculum and extravasation of irrigation fluid into the leg compartments precluded an arthroscopic approach, as further joint distension could exacerbate soft‐tissue edema and compartment syndrome.^12^

A combination of arthroscopic and medial approach for knee dislocation with a medial or lateral‐sided injury has been described.^7^, ^13^, ^14^ A single midline parapatellar approach allowed direct visualization of both femoral condyles and tibial plateaus, facilitating anatomic identification of ligament footprints while protecting neurovascular structures. It also permitted simultaneous reconstruction of cruciate, collateral, and patellofemoral ligaments using a limited number of tunnels and grafts. In contrast, the incision for the midline approach is applicable for a potential future arthroplasty without risk of skin necrosis, which we fear in the existence of multiple previous incisions^15^ (Table 2).

**TABLE 2:** Advantages and Disadvantages of the Open Midline Approach Compared With Arthroscopic Multiligament Reconstruction

Advantages	Disadvantages
Provides excellent exposure for simultaneous reconstruction of cruciate, collateral, and patellofemoral ligaments	Larger incision and potential for slower wound healing compared with arthroscopic portals
Faster procedure and acceleration of the procedure to perform all the reconstructions in a single stage	Greater postoperative pain and stiffness may occur if early rehabilitation is delayed
Avoids excessive arthroscopic fluid extravasation and risk of compartment syndrome in capsular rupture cases	Increased soft‐tissue dissection compared with the arthroscopic manner
Facilitates identification of anatomic landmarks and prevention of tunnel convergence	
Similar approach for a possible future arthroplasty	

The use of common femoral tunnels for cruciate and collateral ligaments prevented tunnel convergence and reduced the number of tunnels in the distal femur, simplifying graft passage and maintaining anatomic orientation. Application of common femoral tunnels has recently been described for ACL and FCL reconstruction^16^ and PCL and MCL reconstruction,^17^ separately. Sequential graft fixation at specific flexion angles (PCL‐MCL at 90°, ACL‐PLC in extension, and MPFL in 30°) ensured restoration of physiologic tensioning patterns.

## DECLARATION OF GENERATIVE AI AND AI‐ASSISTED TECHNOLOGIES IN THE WRITING PROCESS

During the preparation of this work, the authors used ChatGPT to assist with grammar and language editing. Lovevoice AI Voice Generator was used to generate the narration for the accompanying video. After using this tool/service, the authors reviewed and edited the content as needed and take full responsibility for the content of the published article.

## DISCLOSURES

The author (R.F.L.) declares the following financial interests/personal relationships which may be considered as potential competing interests: R.F.L reports a relationship with Smith & Nephew that includes: consulting or advisory and funding grants; reports a relationship with Elsevier that includes: funding grants; reports a relationship with Arthroscopy Association of North America that includes: funding grants; reports a relationship with American Orthopaedic Society for Sports Medicine that includes: funding grants; reports a relationship with Arthrex that includes: funding grants; is a Consultant for OSSUR, Royalties from Ossur. The other authors (S.J., A.A.) declare that they have no known competing financial interests or personal relationships that could have appeared to influence the work reported in this paper.
